# Prevalence and antibiotic resistance of *Salmonella* in organic and non-organic chickens on the Eastern Shore of Maryland, USA

**DOI:** 10.3389/fmicb.2023.1272892

**Published:** 2024-01-04

**Authors:** Anuradha Jeewantha Punchihewage-Don, Jurgen Schwarz, Abdirahman Diria, John Bowers, Salina Parveen

**Affiliations:** ^1^Department of Agriculture, Food and Resource Sciences, University of Maryland Eastern Shore, Princess Anne, MD, United States; ^2^U.S. Food and Drug Administration, College Park, MD, United States

**Keywords:** *Salmonella*, prevalence, antibiotic, resistance, virulence genes

## Abstract

**Introduction:**

*Salmonella* infections have been intensely increasing and becoming a universal public health crisis. This study investigated the prevalence of *Salmonella* in organic and non-organic chickens and the antimicrobial resistance profiles and virulence genes (*inv*A, *pag*C, and *spv*C) in recovered *Salmonella* isolates.

**Methods:**

Whole chicken carcasses [organic (*n* = 240) and non-organic (*n* = 240)] were obtained monthly for 1 year (*n* = 480) from a retail store on the Eastern Shore of Maryland. *Salmonella* isolation and identification were conducted by following the whole carcass enrichment method recommended by USDA-FSIS. Confirmed *Salmonella* isolates (organic *n* = 76; non-organic *n* = 137) were serotyped and tested for antibiotic susceptibility and virulence genes using standard methods.

**Results:**

Forty-nine percent (237/480) of the carcasses were positive for *Salmonella*. Organic and non-organic positivity rates were 37.1 and 61.8%, respectively. A significantly higher *Salmonella* contamination was observed in non-organic chickens (*p* < 0.05). The most common serovars were *Salmonella* Kentucky (47%), *S. Infantis* (35%), *S*. Enteritidis (6%), *S*. *Typhimurium* (5%), and *S*. Blockley (4%). Isolates were frequently resistant to at least one antibiotic (91.24%) or multidrug resistant (45.54%). Resistance was observed to tetracycline (82.8%), minocycline (42.3%), nitrofurantoin (40.3%), cefazolin (38.3%), ampicillin (32.1%), and ceftriaxone (26%). All isolates were susceptible to fluoroquinolone, carbapenem, and glycylcycline. The majority of isolates (99.1%) possessed at least one of three virulence genes of concern and 4.2% tested positive for all three. Ninety-five, 89, and 6.6% of isolates contained *inv*A, *pag*C, and *spv*C genes, respectively. The *spv*C gene was not detected in serovars recovered from organic chickens though 92% and 82% of isolates were positive for *inv*A and *pag*C. The frequency of *Salmonella* recovered from non-organic chickens possessing *inv*A, *pag*C, and *spv*C genes were 97.1, 89.8, and 10.2%, respectively. Detection of *inv*A and *pag*C genes showed no significant difference (*p* > 0.05) between organic and non-organic chickens but a significantly higher *spv*C gene (*p* < 0.05) was detected in non-organic chickens due to the majority of *S*. Enteritidis (92.3%) exclusively recovered from non-organic chicken carried *spv*C gene.

**Discussion:**

This study reveals a high prevalence of *Salmonella* in both organic and non-organic chickens, which exhibit resistance to vital antibiotics and carry virulence genes, thereby creating a potential risk of salmonellosis.

## 1 Introduction

Among foodborne pathogens, *Salmonella* is recognized as a prominent and hazardous foodborne pathogen which is often associated with chickens and the causative agent of the zoonotic disease salmonellosis. Salmonellosis is the second leading foodborne illness in the United States, after norovirus infection (FDA, 2020). In the United States, *Salmonella* is responsible for approximately 1.35 million illnesses, 26,500 hospitalizations, and 420 deaths per annum (CDC, 2023). People get sick by consuming undercooked chicken/poultry products and any other foods that are contaminated by raw chicken or its juices (CDC, 2022). Pathogenesis of *Salmonella* requires the action of multiple virulence factors including *inv*A, *pag*C, and *spv*C to invade, survive, and proliferate in the host. *Salmonella* containing these virulence genes have the potential to cause human illness (Olah et al., 2005; Mohamed et al., 2014). Therefore, investigating virulence factors is crucial for understanding and preventing *Salmonella*-induced foodborne illnesses.

Several poultry-related *Salmonella* outbreaks have been occurring every year in the United States (Punchihewage-Don et al., 2022). Antimicrobial resistance of *Salmonella* is becoming a significant concern for public health in the United States (CDC, 2019; Marchello et al., 2020). Antimicrobials can mitigate the outcome of an infection by destroying or suppressing the growth of pathogens (bacteria, parasites, viruses, and fungi). Antibiotics are medicines that can be used to treat bacterial infections by killing the bacteria (bactericidal) or preventing their multiplication (bacteriostatic) (Punchihewage-Don et al., 2022; World Health Organization [WHO], 2023). Patients with severe *Salmonella* infections are treated with fluoroquinolones (ciprofloxacin), third-generation cephalosporins (ceftriaxone), and macrolides (azithromycin); however, fewer antibiotics are available to treat these severe cases because of increasing AMR. During the past decade, an increasing trend of resistance to medically important antibiotics such as ceftriaxone, ciprofloxacin (non-susceptible), and azithromycin (decreased susceptibility) in non-typhoid *Salmonella* was observed (CDC, 2018, 2019).

Despite the risk of *Salmonella* contamination, chicken is the most frequently consumed meat in the United States (CDC, 2022). According to the National Organic Program (NOP), organically raised chickens must be fed 100% organic feed and no usage of antibiotics, added growth hormones, mammalian or avian byproducts, or other prohibited feed ingredients. Organically raised chickens should also have year around access to the outdoors except during inclement weather conditions (eCFR, 2000; Bailey and Cosby, 2005; USDA-Agricultural Marketing Service, 2013, 2015). In addition, chickens that are treated with antibiotics are prohibited from being sold under the “organic” label (eCFR, 2000). On the other hand, non-organic (conventional) chicken production is a more common chicken farming practice than organic farming and the majority (99%) of the total poultry production in the U.S. comes from non-organic farms (University of Georgia Extension, 2022). Non-organic chickens are fed commercial feed that may contain antimicrobials and dietary supplements. According to the poultry production and value summary, in 2021 more than five billion pounds of broiler chickens were produced in Delaware, Maryland, and Virginia (Delmarva). This is approximately 9% of the total chicken production in the United States and worth over $2.7 billion (USDA-National Agricultural Statistics Service, 2022). The Delmarva peninsula is recognized as the pioneer of chicken processing because the first broiler processing plant in the United States was established in Delaware in 1937 (Constance, 2008).

In order to mitigate foodborne illnesses caused by *Salmonella*, reducing *Salmonella* in food and monitoring the prevalence of resistant strains are important. A few studies were conducted on the prevalence and antimicrobial resistance of *Salmonella* in chicken a few years ago (Parveen et al., 2007; Mazengia et al., 2014; Nguyen et al., 2016). However, little information is available on the prevalence and antimicrobial resistance of *Salmonella* in organic and non-organic chickens (Mak et al., 2022) on the Eastern Shore of Maryland. Therefore, this study was undertaken to determine the prevalence of *Salmonella* in organic and non-organic chickens and investigate antimicrobial resistance profiles and virulence properties of recovered isolates.

## 2 Materials and methods

### 2.1 Sample collection

The sample collection and processing were carried out according to USDA-FSIS (2019) from March 2019 to February 2020. Whole chicken carcasses (organic and non-organic) were obtained monthly from a retail store in the Eastern Shore area, Maryland. A retail store was selected based on the affordability and availability of whole chicken carcasses. During each sampling, 20 organic and 20 non-organic carcasses were collected. These organic and non-organic chickens belonged to two different brands based on their farming practices. All samples were placed in coolers with ice and transported to the food microbiology laboratory at the University of Maryland Eastern Shore within an hour of collection. All the bacterial media utilized in this study were purchased from Becton Dickinson, Sparks, MD, USA unless otherwise specified.

### 2.2 Sample processing

In brief, each carcass was placed in a 4 L sterile plastic stomacher bag (Thermo Fisher Scientific, Hampton, NH, USA). Then sterile buffered peptone water (500 mL) (BPW) was added to the interior and exterior surfaces of each carcass, and the carcass in the bag was shaken vigorously for 60 s. The bag containing the whole carcass and rinse solution were incubated at 37°C for 24 h. *Salmonella enterica* serovar Typhimurium (H_2_S positive) was used as a positive control and sterile BPW was used as a negative control with each batch of samples. After incubation, the sample was screened for *Salmonella* using the BAX system, a commercial PCR-based system (Qualicon Diagnostic, Camarillo, CA, USA). The BAX testing was carried out according to the manufacturer’s guidelines. Samples positive for *Salmonella* were added (0.1 mL) into 10 mL of Rappaport Vassiliadis (RV) broth tubes and the tubes were incubated at 42°C for 24 h. Enriched samples were streaked onto Xylose Lysine agar supplemented with Tergitol 4 (XLT4) and incubated at 37°C for 24 h. After incubation, isolated presumptive *Salmonella* colonies (black centered) were randomly selected and inoculated into Tryptic Soy Broth (TSB) and incubated at 37°C for 24 h to preserve them for further analysis. The incubated samples were centrifuged at 5,000 rpm for 5 min. The remaining pellet was resuspended in TSB with 25% glycerol and stored at −80°C for further analysis (Parveen et al., 2007). Presumptive *Salmonella* isolates were biochemically confirmed using triple sugar iron agar (TSI) and lysine iron agar (LIA) slants (Andrews et al., 2023).

### 2.3 Serotyping of *Salmonella*

All *Salmonella* isolates were serotyped using standard methods at the USDA National Veterinary Services Laboratories (NVSL). Briefly, the isolates were subjected to molecular typing using the xMAP *Salmonella* serotyping assay and classical serotyping using standardized animal antisera to test for the lipopolysaccharide (O antigen) and the flagellar proteins (H antigens) in accordance with the methods described by Edwards and Ewing (Ewing, 1986). Then, the serotypes were designated according to the Kauffmann-White Scheme (Grimont and Weill, 2007).

### 2.4 Antimicrobial resistance of *Salmonella* in organic and non-organic chickens

*Salmonella* isolates were tested for antimicrobial susceptibilities to a panel of 24 antimicrobials of veterinary and human health importance using Sensititre^®^ antimicrobial susceptibility plates following the manufacturer’s instructions (Thermo Fisher Scientific, Hampton, NH, USA). *Escherichia coli* ATCC 25922, and *Pseudomonas aeruginosa* ATCC 27853 were used as controls. The Minimum Inhibitory Concentrations (MICs) were determined as the lowest concentration of an antimicrobial that completely inhibits the growth of bacteria according to the Clinical and Laboratory Standards Institute [CLSI] (2016). The types and ranges of concentrations of the antibiotics are shown in Table 1.

**TABLE 1:** Antibiotic types and range of concentrations.

Antibiotic	Abbreviation	Range of concentration (μ g/mL)	Interpretive categories and MIC breakpoints, (μ g/mL)			
			≤ S*	SDD*	*I* *	≥R*
Ampicillin^1^	AMP	8–16	8	–	16	32
Amikacin^1^	AMI	8–32	16	–	32	64
Ampicillin-sulbactam	A/S2	4/2–16/8	8/4	–	16/8	32/16
Aztreonam^1^	AZT	1–16	4	–	8	16
Cefazolin	FAZ	1–16	2	–	4	8
Cefepime^1^	FEP	2–16	2	4–8	–	16
Ceftazidime^1^	TAZ	1–16	4	–	8	16
Ceftazidime-avibactam	CZA	2/4–16/4	8/4	–	–	16/4
Ceftolozane-tazobactam	C/T	2/4–16/4	2/4	–	–	8/4
Ceftriaxone^1^	AXO	0.5–32	1	–	2	4
Ciprofloxacin^1^	CIP	0.5–2	0.06	–	0.12–0.5	1
Doripenem	DOR	0.5–4	1	–	2	4
Ertapenem	ETP	0.25–8	0.5	–	1	2
Gentamicin^1^	GEN	2–8	4	–	8	16
Imipenem^1^	IMI	1–8	1	–	2	4
Levofloxacin	LEVO	1–8	0.12	–	0.25–1	2
Meropenem	MERO	0.5–8	1	–	2	4
Minocycline	MIN	1–8	4	–	8	16
Nitrofurantoin	NIT	32–64	32	–	64	128
Piperacillin/tazobactam constant^1^	P/T4	8/4–128/32	16/4	–	32/4–16/4	128/4
Tetracycline^2^	TET	4–8	4	–	8	16
Tigecycline	TGC	1–8	0.5	–	–	0.5
Tobramycin	TOB	2–8	4	–	8	16
Trimethoprim/ sulfamethoxazole^2^	SXT	2/38–4/76	2/38	–	–	4/76

*S = Susceptible; SDD = Susceptible-Dose Dependent; I = Intermediate; R = Resistant.

^1^WHO category level I (Critically important to human medicine).

^2^WHO category level II (Highly important to human medicine).

### 2.5 Detection of virulence genes

#### 2.5.1 DNA extraction

DNA extraction of the preserved isolates was done using the InstaGene matrix DNA kit (Bio-Rad, PA, USA) following the manufacturer’s instructions. Briefly, 1–3 confirmed isolated *Salmonella* colonies were suspended with 200 μl of InstaGene matrix and incubated for 30 min at 56°C. The incubated mixture was vortexed for 30 s and proceeded to another incubation at 100°C for 8 min on a heated block. Then, the DNA was separated via centrifugation at 13,200 rpm for 3 min. The supernatant was stored at −20°C for further experiment after measuring DNA concentrations using the Qubit dsDNA HS Assay Kit on a Qubit 3.0 fluorometer (Fisher Scientific, Hampton, NH, USA).

#### 2.5.2 Amplification studies: invA, pagC, and spvC

To determine the presence of the *inv*A, *pag*C, and *spv*C genes a set of primers, shown in Table 2, was used according to Pulkkinen and Miller (1991), Rahn et al. (1992) and Suzuki et al. (1994), respectively. The 50 μL PCR master mix consists of a DNA template (2 μL), deoxynucleotide triphosphates (dATP, dGTP, dCTP, dTTP) at a concentration of 0.25 mM each, MgCl_2_ (2.5 mM), primer (50 pmol/μL), Taq DNA polymerase (1 U), 1 X PCR buffer and distilled water. The amplification parameters were carried out as shown in Table 2 using a PCR system (Applied Biosystems, CA, USA). Thirty cycles of 94°C for 1 min were run to complete the amplification. The amplicon sizes of *inv*A, *pag*C, and *spv*C genes were 284, 318, and 400 base pairs, respectively. For all reactions, *S*. Typhimurium strain LT2 × 3324 containing a recombinant plasmid with *inv*A, *E. coli* DH5-α containing a recombinant plasmid with *spv*C, and *S*. Typhimurium SR 11 × 3337 containing a recombinant plasmid with *pag*C were used as positive controls, while *Escherichia coli* DH5-α (Invitrogen, Carlsbad, CA, USA) was used as a negative control (Olah et al., 2005; Mohamed et al., 2014). The PCR products were separated by electrophoresis in a 1% agarose gel and the gels were stained with GelRed™ (Biotium, Fremont, CA, USA) and viewed with UV light to determine the existence of the PCR products.

**TABLE 2:** PCR primers for amplification.

Primer	Sequence	Denaturation temperature and time	Annealing temperature and time	Extension temperature and time
*inv*A	F–GTGAATTATCGCCACGTTCGG R–TCATCGCACCGTCAAAGGAAC	94°C, 7 min	55°C, 1.5 min	72°C, 1 min (final extension for 5 min)
*pag*C	F–TATGAGGATCACTCTCCGGTA R–ATTCTCCAGCGGATTCATCTA	94°C, 7 min	55°C, 1.5 min	72°C, 1 min (final extension for 7 min)
*spv*C	F–TGGGGCGGAAATACCATCTACAA R–GAACTGAGCGCCCAGGCTAACAC	94°C, 5 min	59°C, 1.5 min	72°C, 1 min (final extension for 7 min)

A Supplementary table, which displays the collection month, serovar, AMR profiles and presence of virulence genes for each isolate, can be found in Supplementary Table 1.

### 2.6 Statistical analysis

The statistical significance of differences in the prevalence of *Salmonella* in organic and non-organic chickens, differences in prevalence of *inv*A, *pag*C, and *spv*C genes, and differences in resistance rate between *Salmonella* serovars and chicken types for each antimicrobial agent tested were determined using Fisher’s exact test. An alpha level of 0.05 was considered the minimum level for statistical significance and, consistent with a per-comparison error rate control approach, *p*-values where unadjusted for the total number of pairwise comparisons. All statistical analyses were performed using R version 4.0.2 (R Core Team, 2021).

## 3 Results and discussion

### 3.1 Prevalence of *Salmonella*

A total of 480 whole chicken carcasses (240 organic and 240 non-organic) were collected during the sampling period (March 2019 to February 2020). Out of 480 whole chicken carcasses, 237 carcasses (49.38%) tested positive for *Salmonella* through molecular screening of primary enrichment. Subsequently, 213 *Salmonella* isolates were cultured, confirmed, and subjected to further testing. Twenty-four *Salmonella* isolates that did not produce typical black-centered colonies during culture confirmation were not chosen for further experiments. According to the present results, 89 (37.08%) and 148 (61.67%) of organic and non-organic chicken carcasses were positive for *Salmonella*, respectively. The results indicated a significantly higher *Salmonella* contamination among non-organic chickens compared to the organic chickens (*p* < 0.05). Contrary to the present results other investigators reported a higher rate of *Salmonella* prevalence in organic chickens (Bailey and Cosby, 2005; Cui et al., 2005). In addition, Lestari et al. (2009) did not find a significant difference between the prevalence of *Salmonella* in organic and non-organic chickens isolated from Louisiana retail stores. Compared to Lestari et al. (2009), the present study has a higher detection rate of *Salmonella*. This might be due to the use of the USDA-FSIS recommended Whole Carcass Enrichment method (WCE). In this method, the entire carcass is subjected to incubation for 24 h at 37°C after vigorously mixing with primary enrichment buffer (BPW) and it helps to proliferate loosely and firmly attached *Salmonella* and increase detection rate (Cox et al., 2014; USDA-FSIS, 2019). A previous study that used the WCE method for the detection of *Salmonella* also showed a high prevalence of *Salmonella* in chickens (Parveen et al., 2007).

**FIGURE 1:**
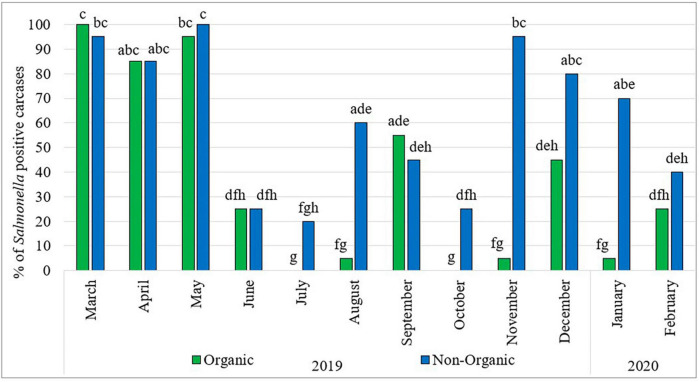
Prevalence of *Salmonella* in organic and non-organic chicken carcasses from March 2019 to February 2020. The Percentage values with different lowercase letters (a-h) are significantly different (*p* < 0.05) amongst comparisons between different months and types of chicken.

Figure 1 shows the prevalence of *Salmonella* in organic and non-organic chicken carcasses during the sampling period. Throughout the 1-year survey period, the prevalence of *Salmonella* fluctuated widely. Comparatively higher rates were observed in the months of March, April and May 2019 in both types of chickens. Thereafter, the prevalence of *Salmonella* in organic chickens was significantly lower and fluctuated during the rest of the sampling period. In the months of July and October, no *Salmonella* was detected in organic chickens and in the months of August, November 2019 and January 2020, only one sample was positive for *Salmonella* in each month. In the case of non-organic chickens, significantly lower *Salmonella* prevalence was observed in the months of June to October 2019 and then significantly higher *Salmonella* prevalence was observed in the months of November 2019 to January 2020. In addition, the month of July was the lowest *Salmonella* prevalence (four positive carcasses) recorded in the entire sampling period among non-organic chickens. However, no seasonal effects with regard to *Salmonella* prevalence on chicken carcasses were observed during the study period. These results are consistent with findings reported by Parveen et al. (2007) and Lestari et al. (2009) who did not find a correlation between *Salmonella* prevalence and the season/month of the year.

### 3.2 Distribution of *Salmonella* serotypes

According to the serotyping results of 213 isolates (Table 3), the top five *Salmonella* serovars were *S.* Kentucky (46.95%), *S.* Infantis (34.27%), *S.* Enteritidis (6.1%), *S.* Typhimurium (5.1%), and *S*. Blockley (5.1%). Table 3 also shows the distribution of *Salmonella* serovars recovered from organic and non-organic chicken carcasses. Similar to the present results, Cui et al. (2005) and Lestari et al. (2009) also reported that *S.* Kentucky was the dominant serovar. A previous study conducted by our lab in 2007 to observe *Salmonella* prevalence in pre- and post-chill broiler carcasses also reported that the predominant serovar was *S*. Kentucky followed by *S*. Typhimurium (Parveen et al., 2007). Furthermore, *S*. Enteritidis was associated only with non-organic chickens while *S*. Blockley was recovered only from organic chickens. In addition, *S*. Typhimurium was more prevalent in organic chickens (10.53%) than in non-organic chickens (2.19%). One of the reasons for the higher *S*. Kentucky and *S*. Infantis observation throughout the year may be the cross contaminations that occurred during the processing of chicken carcasses (Parveen et al., 2007). According to the USDA-FSIS (2023), *S*. Infantis has shown an increasing trend in chicken and has emerged as one of the top serotypes in both cecal and product samples. In addition, Siceloff et al. (2022) reported that the prevalence of *S*. Infantis exceeds that of *S*. Kentucky and becoming a predominant *Salmonella* serovar in this region since 2019. Siceloff et al. (2022) also hypothesized that somehow climate or environmental factors are promoting the colonization of poultry by *S*. Infantis or suppressing the growth of other serovars such as *S*. Kentucky. This explains the higher prevalence of *S.* Infantis occurrence in the present study, even though *S*. Kentucky was dominant among the *Salmonella* collection. However, the association of specific serovars with poultry is not fully understood yet.

**TABLE 3:** Distribution of *Salmonella* serovars recovered from organic and non-organic chicken carcasses.

*Salmonella* serovar	Organic % (*n* = 76)	Non-organic % (*n* = 137)	Total % (*n* = 213)
*S*. Kentucky	38.16	51.82	46.95
*S*. Infantis	32.89	35.04	34.27
*S*. Enteritidis	0b	9.49a	6.10
*S*. Typhimurium	10.53a	2.19b	5.16
*S*. Blockley	10.53a	0b	3.76
*S*. Rough O:r:1,5	3.95a	0b	1.41
*S*. Rough O:r:1,7	2.63	0	0.94
*S*. Rough O:r:1,6	1.32	0	0.47
*S*. 4,[5], 12:i:-	0	0.73	0.47
*S*. III 45:z46:-	0	0.73	0.47

^a,b^Percentages for organic vs. non-organic with different lowercase letters (a, b) are significantly different (*p* < 0.05).

### 3.3 Prevalence of antimicrobial resistance *Salmonella*

**FIGURE 2:**
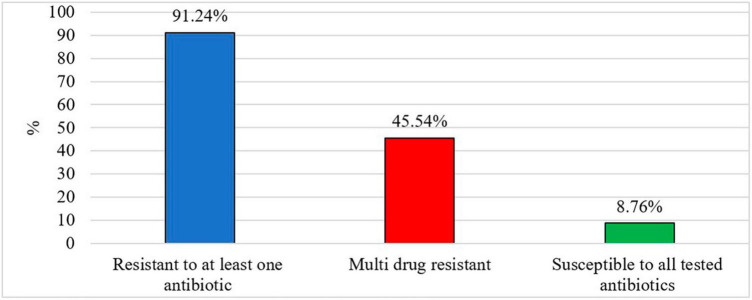
Prevalence of antibiotic resistant *Salmonella* (%).

Out of 213 *Salmonella* isolates, 91.24% were resistant to at least one antibiotic and 8.76% of *Salmonella* isolates were susceptible to all tested antibiotics (Figure 2). Five isolates in each type of chicken showed dose-dependent susceptibility (SDD) to cefepime. Intermediate resistance was observed in 15.78% of isolates recovered in organic chickens [tobramycin (*n* = 1), cefazolin (*n* = 1), and aztreonam (*n* = 10)] and 15.32% of isolates recovered in non-organic chickens [piperacillin/tazobactam constant (*n* = 1), ceftazidime (*n* = 6), ceftriaxone (*n* = 2), cefazolin (*n* = 3), and aztreonam (*n* = 9)]. *Salmonella* isolates were tested for susceptibility to 24 antimicrobial agents belonging to 10 antimicrobial classes that are often prescribed in veterinary and human health. The resistance was often observed to tetracycline (82.8%), minocycline (42.3%), nitrofurantoin (40.3%), cefazolin (38.3%), and ampicillin (32.1%). In this study, ceftriaxone resistant isolates were observed (26%). But all the isolates were susceptible to ciprofloxacin. The frequency of resistant to ceftriaxone in *Salmonella* isolates recovered from the organic and non-organic chickens was 31.6 and 24.1%, respectively. In addition, the frequency of resistance to tobramycin, ampicillin-sulbactam, trimethoprim/sulfamethoxazole, gentamicin, aztreonam, and ceftazidime was 23.5, 11.9, 11.5, 11.0, 10.2, and 3.4%, respectively. Eight percent of *Salmonella* isolates recovered from non-organic chickens were susceptible to all tested antibiotics compared to 10.5% of *Salmonella* isolates recovered from organic chickens. All isolates were susceptible to antibiotic classes of fluoroquinolone, carbapenem, and glycylcycline regardless of the types of chickens (Table 4).

**TABLE 4:** Antimicrobial resistance phenotypes of *Salmonella* isolates recovered from organic and non-organic chickens.

Antibiotic	% of resistant isolates in samples from		
	**Organic (*n* = 76)**	**Non-organic (*n* = 137)**	**Total (*n* = 213)**
Tetracycline	89.5	86.9	82.8
Minocycline	36.8b	53.3a	42.3
Nitrofurantoin	48.7	37.2	40.3
Cefazolin	40.8	40.9	38.3
Ampicillin^2^	35.5	32.8	32.1
Ceftriaxone^1^	31.6	24.1	26.1
Tobramycin	35.5a	14.6b	23.5
Ampicillin-sulbactam	9.2	16.1	11.9
Trimethoprim/sulfamethoxazole^2^	17.1a	7.3b	11.5
Gentamicin	19.7a	3.6b	11.0
Aztreonam	14.5	7.3	10.2
Ceftazidime	0.0b	7.3a	3.4
Amikacin	0.0	0.0	0.0
Piperacillin/tazobactam constant	0.0	0.0	0.0
Ceftolozane-tazobactam	0.0	0.0	0.0
Ceftazidime-avibactam	0.0	0.0	0.0
Levofloxacin	0.0	0.0	0.0
Ciprofloxacin^1^	0.0	0.0	0.0
Doripenem	0.0	0.0	0.0
Ertapenem	0.0	0.0	0.0
Imipenem	0.0	0.0	0.0
Meropenem	0.0	0.0	0.0
Cefepime	0.0	0.0	0.0
Tigecycline	0.0	0.0	0.0

^1^Ceftriaxone and ciprofloxacin are first-line antimicrobial agents used for the treatment of complicated or invasive salmonellosis.

^2^Ampicillin and trimethoprim-sulfamethoxazole are now considered second-line drugs to treat *Salmonella* (FDA, 2022).

^a,b^Percentages for organic vs. non-organic with different lowercase letters (a, b) are significantly different (*p* < 0.05).

*Salmonella* has the capability to adapt to undesirable external environment conditions and it can develop mechanisms to overcome the burden given by the external environment such as antimicrobial drugs (Tomičić et al., 2018). This process is known as antimicrobial resistance. Multiple studies have indicated the prevalence of tetracycline resistance in *Salmonella* (Parveen et al., 2007; Liljebjelke et al., 2017; Velasquez et al., 2018). Previous studies conducted in the United States have similarly documented the resistance of *Salmonella* isolates associated with chickens to cefazolin (Bythwood et al., 2019), ampicillin (Velasquez et al., 2018; Bythwood et al., 2019), and ceftriaxone (Velasquez et al., 2018).

Table 5 shows the distribution of resistant isolates by antibiotic classes and serovars. A significantly higher number (*p* < 0.05) of *S*. Kentucky recovered from non-organic chickens were resistant to ampicillin, ampicillin-sulbactam, and ceftriaxone compared to the *S*. Kentucky isolated from organic chickens. In contrast, a significantly higher number (*p* < 0.05) of *S*. Infantis recovered from organic chickens were resistant to tobramycin, ampicillin, and ceftriaxone. Among *S*. Typhimurium, significantly higher (*p* < 0.05) gentamicin resistance was observed in those recovered from organic chickens. All *S*. Typhimurium recovered from non-organic chickens were resistant to both cefazolin and ampicillin and significantly different from the frequency of resistance of those recovered from organic chickens (*p* < 0.05).

**TABLE 5:** Distribution of resistant isolates by antibiotic classes and serovars (%).

Class	Antibiotic	*S*. Kentucky		*S*. Infantis		*S*. Enteritidis		*S*. Typhimurium	
		**Organic** **(*n* = 29)**	**Non-organic** **(*n* = 71)**	**Organic** **(*n* = 25)**	**Non-organic** **(*n* = 48)**	**Organic** **(*n* = 0)**	**Non-organic** **(*n* = 13)**	**Organic** **(*n* = 8)**	**Non-organic** **(*n* = 3)**
Aminoglycoside	Amikacin	0.0	0.0	0.0	0.0	0.0	0.0	0.0	0.0
	Gentamicin	0.0	0.0	24.0	8.3	0.0	7.7	87.5a	0.0b
	Tobramycin	0.0	2.8	88.0a	37.5b	0.0	0.0	0.0	0.0
β-lactam/β-lactamase inhibitor combination	Piperacillin/tazobactam constant	0.0	0.0	0.0	0.0	0.0	0.0	0.0	0.0
	Ceftolozane-tazobactam	0.0	0.0	0.0	0.0	0.0	0.0	0.0	0.0
	Ampicillin-sulbactam	3.4b	22.5a	20.0	12.5	0.0	0.0	0.0	0.0
	Ceftazidime-avibactam	0.0	0.0	0.0	0.0	0.0	0.0	0.0	0.0
Fluoroquinolone	Levofloxacin	0.0	0.0	0.0	0.0	0.0	0.0	0.0	0.0
	Ciprofloxacin	0.0	0.0	0.0	0.0	0.0	0.0	0.0	0.0
Carbapenem	Doripenem	0.0	0.0	0.0	0.0	0.0	0.0	0.0	0.0
	Ertapenem	0.0	0.0	0.0	0.0	0.0	0.0	0.0	0.0
	Imipenem	0.0	0.0	0.0	0.0	0.0	0.0	0.0	0.0
	Meropenem	0.0	0.0	0.0	0.0	0.0	0.0	0.0	0.0
Folate pathway inhibitor	Trimethoprim/ sulfamethoxazole	0.0	0.0	28.0	20.8	0.0	0.0	0.0	0.0
Cephem	Cefepime	0.0	0.0	0.0	0.0	0.0	0.0	0.0	0.0
	Ceftazidime	0.0	14.1	0.0	0.0	0.0	0.0	0.0	0.0
	Ceftriaxone	0.0b	19.7a	72.0a	35.4b	0.0	7.7	0.0	33.3
	Cefazolin	24.1	38.0	68.0	52.1	0.0	7.7	12.5b	100.0a
Penicillins	Ampicillin	10.3b	31.0a	68.0a	39.6b	0.0	7.7	12.5b	100.0a
Tetracycline	Tetracycline	100.0	94.4	100.0	97.9	0.0	15.4	100.0	100.0
	Minocycline	89.7	93.0	4.0	14.6	0.0	0.0	12.5	0.0
Monobactams	Aztreonam	0.0	2.8	20.0	16.7	0.0	0.0	0.0	0.0
Nitrofurantoin	Nitrofurantoin	10.3	5.6	100.0	93.8	0.0	15.4	37.5	0.0
Glycylcycline	Tigecycline	0.0	0.0	0.0	0.0	0.0	0.0	0.0	0.0

Percentages for organic vs. non-organic, by antibiotic and serovar, with different lowercase letters (a, b) are significantly different (*p* < 0.05). Serovars with a prevalence below the 5% threshold were not included in this table.

Unexpectedly, a higher rate of *Salmonella* isolates recovered from organic chickens showed resistance to some antibiotics compared to *Salmonella* from non-organic chickens (Tables 4, 5). Bailey et al. (2020) also reported such a noticeable antimicrobial resistance in *Salmonella* recovered from organic chickens compared to non-organic chickens. A possible reason for this higher rate of prevalence of antibiotic resistance *Salmonella* among organic chickens could be the farming practice; the organic chickens have access to the outdoors where proper biosecurity measures are difficult to provide. Therefore, there is a higher probability to interact with other avian species, insects, and other wild animals. Avian species such as migratory birds, waterfowl, and other animals that have already been exposed to sources of AMR genes (aquatic environments and other anthropogenic sources) may potentially carry and contaminate the organic chickens with the AMR genes over great distances (Allen et al., 2010; Bailey et al., 2020). Another possible reason for this higher rate of AMR *Salmonella* isolates may be contaminants or antibiotic residues which have been on site before the conversion of a conventional farm to an organic farm (Pesciaroli et al., 2020). One of the requirements for poultry products, that are to be labeled as organic, the poultry must be subjected to organic management from at least the second day of life (eCFR, 2000). But, Pesciaroli et al. (2020) reported that AMR bacteria can be present in 1-day-old chickens due to vertical transmission from the parent and/or contamination at the hatchery.

### 3.4 Antibiotic resistance profiles and MDR *Salmonella*

Sixty resistance profiles were observed in *Salmonella* isolates recovered from both organic and non-organic chickens (Table 6). Twenty-five resistance profiles were observed in *Salmonella* isolates recovered from organic chickens while 50 resistance profiles were detected in *Salmonella* isolates recovered from non-organic chickens. The most common resistance profile was tetracycline-minocycline (27.2%) followed by tetracycline-nitrofurantoin (8.5%) in both types of chickens. The prominent profile among the isolates recovered from the organic chickens was TET-MIN (25.0%) followed by TET-FAZ-NIT-AMP-AXO-TOB (9.2%). The prevalence of the TET-FAZ-NIT-AMP-AXO-TOB-AZT-SXT resistance profile was significantly higher (*p* < 0.05) in *Salmonella* from organic chickens (6.6%) compared to non-organic chickens. The prevalence of the TET-NIT-TOB resistance profile was also significantly higher (*p* < 0.05) in *Salmonella* recovered from organic chickens than in non-organic chickens. One *S*. Infantis isolated from organic chickens showed resistance to 11 antibiotics (TET-FAZ-NIT-MIN-AMP-AXO-AS2-TOB-AZT-SXT-GEN). In addition, two *S*. Infantis and an *S*. Rough_O:1:1,5 were resistant to the profile containing ten antibiotics (TET-FAZ-NIT-AMP-AXO-AS2-TOB-AZT-SXT-GEN). Regarding non-organic chickens, the most common antibiotic resistance profile was TET-MIN (28.5%). The prevalence of the profile containing tetracycline and nitrofurantoin (11.7%) was significantly higher in the isolates recovered from non-organic compared to organic chickens. Five percent of isolates (*S*. Kentucky) were resistant to TET-MIN-FAZ-AMP-AXO-AS2-TAZ. Among the isolates, 3.6% exhibited resistance to TET-MIN-FAZ-AMP profile. One *S*. Infantis isolate (0.7%) showed resistance to ten antimicrobials (TET-FAZ-NIT-AMP-AXO-AS2-TOB-AZT-SXT-GEN) and two *S*. Infantis isolates (1.5%) showed resistance to eight antimicrobials (TET-FAZ-NIT-AMP-AXO-TOB-AZT-GEN). Another *S*. Infantis isolate (0.7%) showed resistance to eight antimicrobials (TET-FAZ-NIT-AMP-AXO-TOB-AZT-SXT). Our results demonstrated a large number of recovered *Salmonella* isolates were resistant to multiple antimicrobials including third-generation cephalosporins (ceftriaxone and ceftazidime). Also, we observed antibiotic resistance profiles with various combinations of antibiotics. Using a higher number of antibiotics (twenty-four different antibiotics) in the AST, we were able to test a large spectrum of antibiotic phenotypes and 60 antibiotic resistance profiles. This is one of the reasons we observed a higher number of antibiotic resistance profiles in our isolates compared to the other studies (Parveen et al., 2007; Lestari et al., 2009).

**TABLE 6:** Antimicrobial resistance profiles.

Resistance profiles	Organic% (*n* = 76)	Non-Organic% (*n* = 137)	Total% (*n* = 213)
TET-FAZ-NIT-MIN-AMP-AXO-AS2-TOB-AZT-SXT-GEN	1.3	0.0	0.5
TET-FAZ-NIT-AMP-AXO-AS2-TOB-AZT-SXT-GEN	3.9	0.7	0.9
TET-FAZ-NIT-AMP-AXO-TOB-AZT-SXT-GEN	1.3	0.0	0.5
TET-FAZ-NIT-AMP-AXO-TOB-AZT-GEN	0.0	1.5	0.9
TET-FAZ-NIT-AMP-AXO-TOB-AZT-SXT	6.6a	0.7b	2.8
TET-FAZ-NIT-AMP-AXO-TOB-SXT-GEN	1.3	0.0	0.5
TET-MIN-FAZ-AMP-AXO-AS2-TAZ	0.0	5.1	3.3
TET-FAZ-NIT-AMP-AXO-TOB-AZT	1.3	0.7	0.9
TET-FAZ-NIT-AMP-AXO-TOB-SXT	2.6	0.7	1.4
TET-NIT-AMP-AXO-TOB-AS2-AZT	1.3	0.0	0.5
TET-MIN-FAZ-AMP-AXO-AS2	0.0	1.5	0.9
TET-MIN-FAZ-NIT-AMP-AS2	1.3	0.7	0.9
TET-FAZ-NIT-AMP-AXO-TOB	9.2	2.9	5.2
TET-NIT-AMP-TOB-AXO-AS2	1.3	0.0	0.5
TET-MIN-FAZ-AMP-AS2	0.0	2.2	1.4
TET-FAZ-NIT-AMP-AXO	1.3	0.7	0.9
TET-MIN-FAZ-AMP	1.3	3.6	2.8
TET-MIN-NIT-GEN	1.3	0.0	0.5
TET-NIT-TOB-SXT	0.0	2.9	1.9
TET-NIT-TOB-GEN	2.6	0.0	0.9
TET-NIT-FAZ-AXO	1.3	0.0	0.5
TET-MIN-FAZ	5.3	0.7	2.3
TET-MIN-NIT	1.3	1.5	1.4
TET-FAZ-NIT	0.0	2.2	1.4
TET-FAZ-AMP	2.6	1.5	1.9
TET-NIT-GEN	2.6	0.7	1.4
TET-NIT-TOB	5.3a	0.0b	1.9
TET-MIN	25.0	28.5	27.2
TET-NIT	2.6b	11.7a	8.5
Other*	7.9	21.2	16.0
Susceptible to all tested antimicrobials	10.5	8.0	8.9

*Other- Resistance profiles which are lower than 1% of both organic and non-organic chicken.

Percentages for organic vs. non-organic with different lowercase letters (a, b) are significantly different (*p* < 0.05).

AMP = Ampicillin; AS2 = Ampicillin-sulbactam; AZT = Aztreonam; FAZ = Cefazolin; TAZ = Ceftazidime; AXO = Ceftriaxone; GEN = Gentamicin; MIN = Minocycline; NIT = Nitrofurantoin; TET = Tetracycline; TOB = Tobramycin; SXT = Trimethoprim/sulfamethoxazole.

After exposure to misused/over-used antibiotics residuals for extended periods, *Salmonella* gains resistance to an antibiotic, it eventually suppresses the actions of the combinations of drugs, leading to the development of Multi Drug Resistance (MDR) (Hetta et al., 2023). In this study, we considered MDR as resistance to at least one agent in three or more antimicrobial classes (Magiorakos et al., 2012; Sapkota et al., 2014). In the present study, we observed MDR in 45.54% of recovered *Salmonella* isolates (Figure 2). Sixty isolates in non-organic chickens (43.80%) and 37 isolates in organic chickens (48.68%) were shown MDR for tested antimicrobial classes. Moreover, a lower prevalence of MDR was observed in *S*. Kentucky (10.35%) isolated from organic chickens compared to *S*. Kentucky (33.8%) in non-organic chickens. In contrast, *S*. Infantis in organic chickens showed a higher prevalence of MDR (96%) compared to *S*. Infantis in non-organic chickens (64.6%). All three isolates of *S*. Typhimurium showed MDR in non-organic chickens and 50% of *S*. Typhimurium isolates showed MDR in organic chickens. In addition, 15.39% of *S*. Enteritidis were MDR. In the present study, we did not observe Extensively Drug Resistant (XDR; resistant to at least one agent in all antimicrobial classes, but susceptible to 1–2 antimicrobial classes) and Pan Drug Resistant (PDR; resistant to all agents in all antimicrobial classes) *Salmonella* isolates among both types of chickens. If *Salmonella* develops its resistance to the multiple drugs that are used to treat severe bacterial diseases in both animals and humans, new effective drugs must be invented to control *Salmonella* infections.

### 3.5 Virulence properties of recovered *Salmonella* isolates

The majority of isolates (99.1%) possessed at least one of the *inv*A, *pag*C, and *spv*C genes. Only two isolates did not possess any of these three genes (*S*. III 45:z46:- and an *S*. Infantis). Among tested isolates, 4.2% were positive for all three virulence genes. Regardless of the type of chickens, the prevalence of *inv*A, *pag*C, and *spv*C genes in *Salmonella* isolates was 95.3, 89.2, and 6.6%, respectively. The prevalence of the *inv*A gene in the *Salmonella* isolates recovered from organic chickens was 92% and the prevalence of *pag*C was 88%. The *spv*C gene was not detected in the *Salmonella* isolates recovered from organic chickens. On the other hand, *inv*A, *pag*C, and *spv*C genes were detected in *Salmonella* recovered from non-organic chickens with a prevalence of 97, 89, and 10%, respectively. A significantly higher prevalence of the *spv*C gene (*p* < 0.05) was observed in non-organic chickens compared to organic chickens (*p* < 0.05). The majority of *spv*C genes were carried by *S*. Enteritidis (92.3%) which was exclusively recovered from non-organic chicken samples. Furthermore, one *S*. 4,[5],12:i:- and one *S*. Kentucky were recovered from non-organic chickens carried the *spv*C gene as well as *inv*A and *pag*C genes (as shown in Table 7). Importantly, 53.8% of *S*. Enteritidis isolates were found to possess all three genes.

**TABLE 7:** The distribution of *inv*A, *pag*C, and *spv*C genes in *Salmonella* isolates recovered from organic and non-organic chicken.

*Salmonella* serovar	Number of serovars recovered	*Salmonella* recovered from organic chicken (%)		
		***inv*A**	***pag*C**	***spv*C**
*S*. Kentucky	29	28 (96.6)	27 (93.1)	0 (0)
*S*. Infantis	25	21 (84)	22 (88)	0 (0)
*S*. Typhimurium	8	8 (100)	5 (62.5)	0 (0)
*S*. Blockley	8	7 (87.5)	7 (87.5)	0 (0)
*S*. Rough_O:r:1,5	3	3 (100)	3 (100)	0 (0)
*S*. Rough_O:r:1,7	2	2 (100)	2 (100)	0 (0)
*S*. Rough_O:r:1,6	1	1 (100)	1 (100)	0 (0)
***Salmonella* serovar**	**Number of serovars recovered**	***Salmonella* recovered from non-organic chicken (%)**		
		***inv*A**	***pag*C**	***spv*C**
*S*. Kentucky	71	70 (98.6)	67 (94.4)	1 (1.4)
*S*. Infantis	48	47 (97.9)	44 (91.7)	0 (0)
*S*. Typhimurium	3	3 (100)	3 (100)	0 (0)
*S*. Enteritidis	13	12 (92.3)	8 (61.5)	12 (92.3)
*S*. 4,[5], 12:i:-	1	1 (100)	1 (100)	1 (100)
*S*. III 45:z46:-	1	0 (0)	0 (0)	0 (0)

These *inv*A, *pag*C, and *spv*C genes are playing a major role in *Salmonella* virulence (Nolan et al., 1995; Olah et al., 2005). The *inv*A gene which is located in the chromosome of *Salmonella* promotes the invasion of the host cell by stimulating the formation of inner and outer membrane proteins (Olah et al., 2005; Mohamed et al., 2014). *Pag*C is another essential chromosomal virulence gene that encodes an outer membrane/envelope protein that promotes survival within macrophages by suppressing bacterial cell division and prolonging the cell cycle (Olah et al., 2005; Xu et al., 2018). The expression of *pag*C gene is activated by *Salmonella* cells as a defensive mechanism against stress conditions inside the host. In unfavorable environmental conditions, *pag*C gene promotes growth under low Mg^2+^ concentrations, resistance to low pH, bile salts, and cationic antimicrobial proteins (Xu et al., 2018). Therefore, *pag*C plays an important role in *Salmonella* virulence, inducing *Salmonella* cells to enter a Viable but Non-Culturable (VBNC) state under unfavorable environmental conditions (Xu et al., 2018). The *spv*C gene which is located on *Salmonella* Typhimurium virulence plasmid, promotes the prolific growth of *Salmonella* in host reticuloendothelial tissues (Olah et al., 2005). Our results indicated a high prevalence of *inv*A and *pag*C among the *Salmonella* isolates recovered from both organic and non-organic chickens compared to *spv*C gene. Olah et al. (2005) and Mohamed et al. (2014) observed that all the tested isolates were positive for the *inv*A and *pag*C but observed low detection of *spv*C gene. It has been suggested that the *inv*A gene is exclusive to the *Salmonella* genome and serves as a distinctive marker, allowing for molecular detection of *Salmonella* (Rahn et al., 1992; Chiu and Ou, 1996; Wolffs et al., 2006; Andrews et al., 2023). However, mutations occurred in the *inv*A gene may not be identifiable through traditional PCR based methods. In addition, the *Salmonella* carrying mutant *inv*A gene showed less virulent (Galán et al., 1992; Ginocchio and Galán, 1995; Darwin and Miller, 1999). According to previous studies, the detection of *spv*C gene is low compared to *inv*A and *pag*C genes (Nolan et al., 1995; Mohamed et al., 2014; Sharma et al., 2019; Tasmin et al., 2019). It has been reported that *spv*C gene is frequently found in a limited number of serovars including *S. Enteritidis*, *S. Typhimurium*, *S. Choleraesuis*, and *S. Dublin* (mostly from infected animals and birds) (Krzyzanowski et al., 2014; Tasmin et al., 2019) and its presence or absence seems to be correlated with the possession of virulence plasmid (Olah et al., 2005). In the present study, the majority of *S.* Enteritidis (*n* = 12), *S.* 4,[5],12:i:- (*n* = 1), and *S.* Kentucky (*n* = 1) had *spv*C gene in their genome. Apparently, in our *Salmonella* collection, *S*. Typhimurium recovered from both types of chickens did not possess *spv* operon. In the present study, we observed a comparatively higher rate of *spv*C gene in the non-organic chickens because the *S.* Enteritidis recovered only from the non-organic chickens and the majority (92.3%) of them carried *spv*C gene. Another possible reason for the lower *spv*C gene detection is the method of DNA extraction. In the present study, we used a DNA extraction method that focused on chromosomal DNA extraction but not specifically designed for plasmids. However, it has been suggested that further research is needed to understand the factors that affect the presence and expression of *spv*C gene in *Salmonella* (Mohamed et al., 2014).

*Salmonella* which carries the *inv*A, *pag*C, and *spv*C genes has the potential to cause foodborne illnesses when ingested with contaminated food (Olah et al., 2005; Mohamed et al., 2014). Four isolates identified in our study that carried all three of these genes showed resistance to multiple drugs. The *S*. Kentucky isolate which had all three genes showed resistance to cefazolin, ampicillin, tetracycline, and minocycline. In addition, *S.* Enteritidis isolates which carried all three genes were resistant to multiple drugs in classes of cephem (ceftriaxone and cefazolin), tetracycline, nitrofurantoin, and penicillin. Moreover, an *S.* Enteritidis isolate was resistant to gentamicin which belongs to the class of aminoglycoside in addition to the above-mentioned antibiotics. Ceftriaxone is one of the important antibiotics that can be used to treat severe *Salmonella* infection (CDC, 2018). If salmonellosis occurs due to ingestion of an MDR *Salmonella* serovar which carries these virulence genes, it would be a challenge to select appropriate antibiotics to control the disease.

## 4 Conclusion

The results of this study demonstrate a high prevalence of *Salmonella* contamination in organic and non-organic chickens and a significant number of these isolates were resistant to commonly used antibiotics. The *Salmonella* isolates recovered from both types of chickens possessed virulence genes and thus have the potential to cause salmonellosis. It will be a challenge to treat a patient who has a severe *Salmonella* infection caused by an MDR *Salmonella* strain. Conscious action must be taken to reduce *Salmonella* contamination throughout the food chain. In order to have a broader knowledge of the topic a large-scale and multi-state-wide study is highly recommended. The result of our study highlighted the importance of educating consumers on safe food handling practices in the home to improve their self-hygiene practices, eliminate cross-contamination and avoid consuming undercooked food.

## Data availability statement

The raw data supporting the conclusions of this article will be made available by the authors, without undue reservation.

## Author contributions

AJP-D: Data curation, Formal analysis, Investigation, Methodology, Software, Visualization, Writing—original draft, Writing—review and editing. JS: Conceptualization, Writing—review and editing, Funding acquisition. AD: Methodology, Writing—review and editing. JB: Formal analysis, Software, Writing—review and editing. SP: Conceptualization, Funding acquisition, Investigation, Project administration, Resources, Supervision, Writing—review and editing, Data curation, Visualization.
