# Supplementary material for: Prevalence and antibiotic resistance of Salmonella in organic and non-organic chickens on the Eastern Shore of Maryland, USA
Source: Front Microbiol. 2024 Jan 4;14:1272892. doi: 10.3389/fmicb.2023.1272892 (PMC10794514; doi:10.3389/fmicb.2023.1272892)
Supplement: Supplementary file 1 [file Table_1.DOCX]

Table S1. Summary of *Salmonella* isolation and serovar identification based on the month of sample collection

| Collection Month | Sample ID | Type of Chicken | *Salmonella* Serovar | Resistance to the Antibiotic | | | | | | | | | | | | Virulence Genes | | |
| --- | --- | --- | --- | --- | --- | --- | --- | --- | --- | --- | --- | --- | --- | --- | --- | --- | --- | --- |
|  |  |  |  | TET | MIN | NIT | AMP | TOB | GEN | AZT | AS2 | SXT | AXO | FAZ | TAZ | *invA* | *pagC* | *spvC* |
| March 2019 | 1 | O | Kentucky | R | R |  |  |  |  |  |  |  |  |  |  | + | - | - |
|  | 2 | O | Blockey |  |  |  |  |  |  |  |  |  |  |  |  | + | + | - |
|  | 3 | O | Infantis | R |  | R |  |  |  |  |  |  | R | R |  | + | + | - |
|  | 4 | O | Infantis | R |  | R |  | R | R |  |  |  |  |  |  | + | + | - |
|  | 5 | O | Infantis | R |  | R |  | R |  |  |  |  |  |  |  | + | + | - |
|  | 6 | O | Blockey |  |  |  |  |  |  |  |  |  |  |  |  | - | + | - |
|  | 7 | O | Blockey |  |  |  |  |  |  |  |  |  |  |  |  | + | + | - |
|  | 8 | O | Blockey |  |  |  |  |  |  |  |  |  |  |  |  | + | + | - |
|  | 9 | O | Blockey |  |  |  |  |  |  |  |  |  |  |  |  | + | + | - |
|  | 10 | O | Infantis | R |  | R |  | R |  |  |  |  |  |  |  | + | + | - |
|  | 11 | O | Infantis | R |  | R |  |  |  |  |  |  |  |  |  | + | + | - |
|  | 12 | O | Infantis | R |  | R |  | R |  |  |  |  |  |  |  | + | + | - |
|  | 13 | O | Infantis | R |  | R | R | R |  |  |  |  | R | R |  | + | + | - |
|  | 14 | O | Blockey |  |  |  |  |  |  |  |  |  |  |  |  | + | - | - |
|  | 15 | O | Infantis | R |  | R | R | R |  |  |  |  | R | R |  | - | - | - |
|  | 16 | O | Infantis | R |  | R | R | R |  |  |  |  | R | R |  | + | + | - |
|  | 17 | O | Infantis | R |  | R | R | R |  |  |  |  | R | R |  | + | + | - |
|  | 18 | O | Infantis | R |  | R | R |  |  |  |  |  | R | R |  | + | + | - |
|  | 19 | O | Infantis | R |  | R | R | R |  |  |  |  | R | R |  | - | + | - |
|  | 20 | O | Infantis | R |  | R | R | R |  | R | R |  | R |  |  | - | + | - |
|  | 21 | N | Infantis | R |  | R | R |  |  | I |  |  | R | R |  | + | - | - |
|  | 22 | N | Infantis | R |  | R |  |  |  |  |  |  |  |  |  | - | + | - |
|  | 23 | N | Infantis | R |  | R |  |  |  |  |  |  |  |  |  | + | + | - |
|  | 24 | N | Kentucky | R | R |  | R |  |  |  | R |  | R | R | R | + | + | - |
|  | 26 | N | Kentucky | R | R |  | R |  |  |  | R |  | R | R | I | + | + | - |
|  | 27 | N | Infantis | R | R | R | R | R |  | I |  |  | R | R |  | + | + | - |
|  | 28 | N | Infantis | R |  | R |  |  |  |  |  |  |  |  |  | + | + | - |
|  | 29 | N | Infantis | R |  | R |  |  |  |  |  |  |  |  |  | + | + | - |
|  | 30 | N | Infantis | R |  | R |  |  |  |  |  |  |  |  |  | + | + | - |
|  | 31 | N | Kentucky | R | R |  |  |  |  |  |  |  |  |  |  | - | + | - |
|  | 32 | N | Kentucky | R | R |  | R |  |  |  |  |  | R | R | I | + | - | - |
|  | 33 | N | Infantis | R |  | R | R | R | R | R |  |  | R | R |  | + | + | - |
|  | 34 | N | Infantis | R |  | R | R | R |  | I |  |  | R | R |  | + | + | - |
|  | 35 | N | Infantis | R |  | R | R | R |  | I |  |  | R | R |  | + | + | - |
|  | 36 | N | Infantis | R |  | R |  |  |  |  |  |  |  |  |  | + | - | - |
|  | 37 | N | Infantis | R |  | R | R | R |  | I |  |  | R | R |  | + | + | - |
|  | 38 | N | Kentucky | R | R |  |  |  |  |  |  |  |  |  |  | + | - | - |
|  | 39 | N | Kentucky | R | R |  |  |  |  |  |  |  |  |  |  | + | + | - |
|  | 40 | N | Infantis | R |  | R | R | R |  | I |  |  | R | R |  | + | + | - |
| April 2019 | 41 | O | Rough_O:1:1,5 | R |  | R | R | R | R | R | R | R | R | R |  | + | + | - |
|  | 43 | O | Infantis | R |  | R | R | R | R |  |  | R | R | R |  | + | + | - |
|  | 44 | O | Rough_O:1:1,5 | R |  | R | R | R |  | R |  | R | R | R |  | + | + | - |
|  | 45 | O | Infantis | R |  | R | R | R |  |  | R |  | R | R |  | - | + | - |
|  | 46 | O | Infantis | R |  | R | R | R |  |  |  |  | R | R |  | + | + | - |
|  | 47 | O | Rough_O:1:1,5 | R |  | R | R | R |  | R |  | R | R | R |  | + | + | - |
|  | 49 | O | Rough_O:1:1,6 | R |  | R | R | R |  | R |  | R | R | R |  | + | + | - |
|  | 50 | O | Rough_O:1:1,7 | R |  | R | R | R | R | R |  | R | R | R |  | + | + | - |
|  | 51 | O | Infantis | R |  | R | R | R |  | R |  | R | R | R |  | + | + | - |
|  | 53 | O | Rough_O:1:1,7 | R |  | R | R | R |  | R |  | R | R | R |  | + | + | - |
|  | 54 | O | Infantis | R |  | R | R | R |  |  |  | R | R | R |  | + | - | - |
|  | 57 | O | Infantis | R |  | R | R | R |  |  |  | R | R | R |  | + | - | - |
|  | 58 | O | Kentucky | R |  |  |  |  |  |  |  |  |  |  |  | + | + | - |
|  | 60 | O | Infantis | R |  | R | R | R |  |  |  |  | R | R |  | + | + | - |
|  | 61 | N | Typhimurium | R |  |  | R |  |  |  |  |  |  | R |  | + | + | - |
|  | 62 | N | Infantis | R |  | R |  |  |  |  |  |  |  | R |  | + | + | - |
|  | 63 | N | Typhimurium | R |  |  | R |  |  |  |  |  |  | R | I | + | + | - |
|  | 64 | N | Infantis | R |  | R |  |  |  |  |  |  |  |  |  | + | + | - |
|  | 65 | N | Infantis | R |  | R |  |  |  |  |  |  |  |  |  | + | + | - |
|  | 66 | N | Infantis | R |  | R | R |  |  |  |  |  |  | R |  | + | + | - |
|  | 67 | N | Infantis | R |  | R | R | R |  | I |  | R | R | R |  | + | + | - |
|  | 68 | N | Infantis | R |  | R |  |  |  |  |  |  |  |  |  | + | + | - |
|  | 69 | N | Infantis | R |  | R | R | R |  | R |  | R | R | R |  | + | + | - |
|  | 71 | N | Kentucky | R | R |  |  |  |  |  |  |  |  | R |  | + | + | - |
|  | 72 | N | Infantis | R |  | R | R |  |  |  |  |  |  |  |  | + | + | - |
|  | 73 | N | Infantis | R |  | R |  |  |  |  |  |  |  |  |  | + | + | - |
|  | 75 | N | Infantis | R | R |  | R |  |  |  | R |  | R | R | I | + | + | - |
|  | 76 | N | Kentucky | R | R |  |  |  |  |  |  |  |  |  |  | + | + | - |
|  | 78 | N | Kentucky | R | R |  | R |  |  |  |  |  |  | R |  | + | + | - |
|  | 79 | N | Infantis | R |  | R |  |  |  |  |  |  |  | R |  | + | + | - |
|  | 80 | N | Enteritidis |  |  |  |  |  |  |  |  |  |  |  |  | + | + | + |
| May 2019 | 81 | O | Kentucky | R | R |  |  |  |  |  |  |  |  |  |  | + | + | - |
|  | 82 | O | Kentucky | R | R |  |  |  |  |  |  |  |  |  |  | + | + | - |
|  | 83 | O | Kentucky | R | R |  |  |  |  |  |  |  |  |  |  | + | + | - |
|  | 84 | O | Kentucky | R | R |  |  |  |  |  |  |  |  |  |  | + | + | - |
|  | 85 | O | Kentucky | R | R |  |  |  |  |  |  |  |  |  |  | + | + | - |
|  | 86 | O | Kentucky | R |  |  | R |  |  |  |  |  |  | R |  | + | + | - |
|  | 87 | O | Kentucky | R | R |  |  |  |  |  |  |  |  | R |  | + | + | - |
|  | 88 | O | Kentucky | R | R |  |  |  |  |  |  |  |  | I |  | + | + | - |
|  | 89 | O | Kentucky | R | R |  |  |  |  |  |  |  |  | R |  | + | + | - |
|  | 90 | O | Kentucky | R | R |  |  |  |  |  |  |  |  | R |  | - | + | - |
|  | 91 | O | Kentucky | R | R |  |  |  |  |  |  |  |  |  |  | + | + | - |
|  | 92 | O | Typhimurium | R |  |  |  |  | R |  |  |  |  |  |  | + | + | - |
|  | 93 | O | Kentucky | R | R |  |  |  |  |  |  |  |  |  |  | + | + | - |
|  | 94 | O | Kentucky | R | R |  |  |  |  |  |  |  |  |  |  | + | + | - |
|  | 95 | O | Kentucky | R | R |  |  |  |  |  |  |  |  | R |  | + | + | - |
|  | 96 | O | Blockey |  |  |  |  |  |  |  |  |  |  |  |  | + | + | - |
|  | 97 | O | Blockey |  |  |  |  |  |  |  |  |  |  |  |  | + | + | - |
|  | 99 | O | Kentucky | R | R |  |  |  |  |  |  |  |  |  |  | + | + | - |
|  | 101 | N | Infantis | R |  | R |  |  |  |  |  |  |  |  |  | + | + | - |
|  | 102 | N | Kentucky | R | R |  |  |  |  |  |  |  |  |  |  | + | + | - |
|  | 103 | N | Infantis | R | R |  |  |  |  |  |  |  |  |  |  | + | + | - |
|  | 104 | N | Kentucky | R | R |  |  |  |  |  |  |  |  |  |  | + | + | - |
|  | 107 | N | Infantis | R | R | R |  |  |  |  |  |  |  |  |  | + | + | - |
|  | 109 | N | Kentucky | R | R | R |  |  |  |  |  |  |  |  |  | + | + | - |
|  | 110 | N | Kentucky | R | R |  |  |  |  |  |  |  |  |  |  | + | + | - |
|  | 111 | N | Kentucky | R | R |  |  |  |  |  |  |  |  |  |  | + | + | - |
|  | 112 | N | Kentucky | R | R |  | R |  |  |  | R |  | R | R | R | + | + | - |
|  | 113 | N | Kentucky | R | R |  | R |  |  |  | R |  | R | R | R | + | + | - |
|  | 114 | N | Infantis | R |  | R |  |  |  |  |  |  |  |  |  | + | + | - |
|  | 115 | N | Kentucky | R | R |  |  |  |  |  |  |  |  |  |  | + | + | - |
|  | 117 | N | Kentucky | R | R |  |  |  |  |  |  |  |  |  |  | + | + | - |
|  | 118 | N | Infantis | R |  | R | R | R | R | R |  |  | R | R |  | + | + | - |
|  | 119 | N | Infantis | R |  | R |  |  |  |  |  |  |  |  |  | + | + | - |
|  | 120 | N | Infantis | R |  | R |  |  |  |  |  |  |  |  |  | + | + | - |
| June 2019 | 128 | O | Kentucky | R | R |  |  |  |  |  |  |  |  |  |  | + | + | - |
|  | 131 | O | Kentucky | R |  | R |  |  |  |  |  |  |  |  |  | + | + | - |
|  | 144 | N | Enteritidis |  |  |  |  |  |  |  |  |  |  |  |  | + | - | + |
|  | 146 | N | Infantis | R |  | R |  |  |  |  |  |  |  |  |  | + | + | - |
|  | 151 | N | Infantis | R | R | R |  |  |  |  |  |  |  | R |  | + | + | - |
|  | 153 | N | Kentucky | R | R |  | R |  |  |  |  |  |  | R |  | + | + | + |
|  | 157 | N | Kentucky | R | R |  |  |  |  |  | R |  |  | R |  | + | + | - |
| July 2019 | 181 | N | Infantis | R |  | R |  |  |  |  |  |  |  | R |  | + | + | - |
|  | 184 | N | Infantis |  |  | R |  | R | R |  | R | R | R | R |  | + | + | - |
|  | 190 | N | Kentucky | R | R |  |  |  |  |  |  |  | R | R | R | + | + | - |
|  | 195 | N | Enteritidis |  |  |  |  |  |  |  |  |  |  |  |  | + | - | + |
|  | 199 | N | Infantis | R |  | R |  |  |  |  |  |  |  | I |  | + | + | - |
| August 2019 | 219 | O | Typhimurium | R |  |  |  |  | R |  |  |  |  |  |  | + | - | - |
|  | 221 | N | Kentucky | R | R |  | R |  |  |  | R |  | R | R | R | + | + | - |
|  | 222 | N | Kentucky | R | R |  |  |  |  |  |  |  |  |  |  | + | + | - |
|  | 223 | N | Kentucky | R |  |  |  |  |  |  |  |  |  |  |  | + | + | - |
|  | 225 | N | Kentucky | R | R |  |  |  |  |  |  |  |  |  |  | + | + | - |
|  | 227 | N | Kentucky | R | R |  | R |  |  |  | R |  | R | R | R | + | + | - |
|  | 228 | N | Enteritidis |  |  |  |  |  |  |  |  |  |  |  |  | + | + | + |
|  | 229 | N | III 45:z46:- |  |  |  |  |  |  |  |  |  |  | R |  | - | - | - |
|  | 230 | N | Kentucky | R | R |  |  |  |  |  |  |  |  |  |  | + | + | - |
|  | 233 | N | Enteritidis |  |  |  |  |  |  |  |  |  |  |  |  | + | - | + |
|  | 237 | N | Kentucky | R | R |  |  |  |  |  |  |  |  |  |  | + | + | - |
|  | 238 | N | Kentucky | R | R |  |  |  |  |  |  |  |  |  |  | + | + | - |
|  | 239 | N | Kentucky | R | R |  |  |  |  |  | R |  | R | R | R | + | + | - |
| September 2019 | 241 | O | Kentucky | R | R | R |  |  |  |  |  |  |  |  |  | + | + | - |
|  | 243 | O | Kentucky | R | R |  |  |  |  |  |  |  |  |  |  | + | + | - |
|  | 244 | O | Kentucky | R | R |  |  |  |  |  |  |  |  |  |  | + | + | - |
|  | 247 | O | Kentucky | R | R |  |  |  |  |  |  |  |  |  |  | + | + | - |
|  | 248 | O | Kentucky | R | R |  |  |  |  |  |  |  |  |  |  | + | + | - |
|  | 249 | O | Kentucky | R | R |  |  |  |  |  |  |  |  |  |  | + | + | - |
|  | 250 | O | Kentucky | R | R |  |  |  |  |  |  |  |  |  |  | + | + | - |
|  | 251 | O | Kentucky | R | R | R | R |  |  |  | R |  |  | R |  | + | - | - |
|  | 258 | O | Kentucky | R | R |  | R |  |  |  |  |  |  | R |  | + | + | - |
|  | 260 | O | Kentucky | R | R |  |  |  |  |  |  |  |  |  |  | + | + | - |
|  | 266 | N | Kentucky | R | R |  |  |  |  |  |  |  |  |  |  | + | + | - |
|  | 268 | N | Kentucky | R | R |  | R |  |  |  | R |  | R | R | R | + | + | - |
|  | 269 | N | Kentucky | R | R |  |  |  |  |  |  |  |  |  |  | + | + | - |
|  | 272 | N | Kentucky | R | R |  | R |  |  |  |  |  |  | R |  | + | + | - |
|  | 273 | N | Kentucky | R | R |  | R |  |  |  |  |  |  | R |  | + | + | - |
|  | 277 | N | Kentucky | R |  |  |  |  |  |  |  |  |  | R |  | + | + | - |
|  | 278 | N | Kentucky | R | R |  |  |  |  |  |  |  |  | I |  | + | + | - |
|  | 280 | N | Kentucky | R | R |  |  |  |  |  |  |  |  | I |  | + | + | - |
| October 2019 | 317 | N | Kentucky | R | R |  | R |  |  |  | R |  |  | R |  | + | + | - |
|  | 318 | N | Kentucky |  | R |  | R |  |  | I | R |  | R | R | R | + | + | - |
|  | 319 | N | Kentucky | R | R |  |  |  |  |  |  |  |  |  |  | + | + | - |
|  | 320 | N | Enteritidis |  |  |  |  |  |  |  |  |  |  |  |  | + | + | - |
| November 2019 | 341 | N | Kentucky | R | R |  |  |  |  |  |  |  |  |  |  | + | + | - |
|  | 342 | N | Infantis | R |  | R | R |  |  | R |  |  | R | R |  | + | + | - |
|  | 343 | N | Infantis | R |  | R | R | R |  | R | R |  | R | R |  | + | + | - |
|  | 344 | N | Infantis | R |  | R | R | R |  | R |  |  | R | R |  | + | + | - |
|  | 345 | N | Kentucky |  |  |  | R |  |  |  | R |  | R | R |  | + | + | - |
|  | 347 | N | Kentucky | R | R |  |  |  |  |  |  |  |  |  |  | + | + | - |
|  | 348 | N | Enteritidis |  |  |  | R |  |  |  |  |  | I | R |  | + | + | + |
|  | 349 | N | Kentucky |  | R |  | R |  |  |  | R |  | R | R |  | + | + | - |
|  | 350 | N | Kentucky | R | R |  |  |  |  | R |  |  |  |  |  | + | + | - |
|  | 352 | N | Kentucky |  |  |  |  |  |  |  |  |  |  | R |  | + | + | - |
|  | 353 | N | Kentucky | R | R |  | R |  |  |  |  |  |  |  |  | + | + | - |
|  | 354 | N | Kentucky | R | R |  |  |  |  |  |  |  |  |  |  | + | + | - |
|  | 355 | N | Kentucky | R | R |  | R |  |  |  | R |  | R | R | R | + | + | - |
|  | 356 | N | Kentucky | R | R |  |  |  |  |  |  |  |  |  |  | + | + | - |
|  | 358 | N | Kentucky | R | R | R | R |  |  |  | R |  |  | R | I | + | + | - |
|  | 359 | N | Infantis | R | R | R | R |  |  | R | R |  | R | R |  | + | + | - |
|  | 360 | N | Kentucky | R |  |  | R |  |  |  |  |  |  |  |  | + | + | - |
| December 2019 | 361 | O | Infantis | R |  | R |  | R | R |  |  |  |  |  |  | + | + | - |
|  | 362 | O | Infantis | R |  | R | R | R | R | R | R | R | R | R |  | + | + | - |
|  | 365 | O | Infantis | R |  | R |  | R |  |  |  |  |  |  |  | + | + | - |
|  | 371 | O | Infantis | R |  | R | R | R | R | R | R | R | R | R |  | + | + | - |
|  | 373 | O | Infantis | R | R | R | R | R | R | R | R | R | R | R |  | + | + | - |
|  | 382 | N | Kentucky | R | R | R |  | R |  |  |  |  |  |  |  | + | + | - |
|  | 383 | N | Kentucky | R | R | R |  | R |  | R |  |  | R | R |  | + | + | - |
|  | 384 | N | Kentucky | R | R |  |  |  |  |  |  |  |  |  |  | + | + | - |
|  | 385 | N | Kentucky | R | R |  |  |  |  |  |  |  |  |  |  | + | + | - |
|  | 386 | N | Kentucky | R | R |  |  |  |  |  |  |  |  |  |  | + | + | - |
|  | 387 | N | Enteritidis | R |  | R |  |  |  |  |  |  | R |  |  | + | + | + |
|  | 388 | N | Infantis | R | R |  | R |  |  |  | R |  |  | R |  | + | + | - |
|  | 389 | N | Kentucky | R | R |  | R |  |  |  | R |  |  | R |  | + | + | - |
|  | 390 | N | Kentucky | R | R |  |  |  |  |  |  |  |  |  |  | + | + | - |
|  | 391 | N | Kentucky | R | R |  |  |  |  |  |  |  |  |  |  | + | + | - |
|  | 393 | N | Kentucky | R | R |  |  |  |  |  |  |  |  |  |  | + | + | - |
|  | 394 | N | Kentucky | R | R |  |  |  |  |  |  |  |  |  |  | + | + | - |
|  | 398 | N | Kentucky | R | R |  |  |  |  |  |  |  |  |  |  | + | + | - |
|  | 400 | N | 4,[5], 12:i:- |  |  |  |  |  |  |  |  |  |  |  |  | + | + | + |
| January 2020 | 416 | O | Typhimurium | R |  |  | R |  |  |  |  |  |  | R |  | + | + | - |
|  | 422 | N | Enteritidis |  |  |  |  |  |  |  |  |  |  |  |  | + | + | + |
|  | 423 | N | Infantis | R |  | R |  | R |  |  |  | R |  |  |  | + | + | - |
|  | 424 | N | Kentucky | R | R |  |  |  |  |  |  |  |  |  |  | + | + | - |
|  | 427 | N | Enteritidis |  |  |  |  |  |  |  |  |  |  |  |  | + | - | + |
|  | 428 | N | Kentucky | R | R |  | R |  |  |  |  |  |  | R |  | + | - | - |
|  | 429 | N | Infantis | R |  | R |  | R |  |  |  | R |  |  |  | + | - | - |
|  | 431 | N | Infantis | R |  | R |  | R |  |  |  | R |  |  |  | + | + | - |
|  | 432 | N | Infantis | R |  | R |  | R |  |  |  | R |  |  |  | + | + | - |
|  | 434 | N | Kentucky | R | R |  |  |  |  |  |  |  |  |  |  | + | - | - |
|  | 435 | N | Enteritidis | R |  | R |  |  | R |  |  |  |  |  |  | + | + | + |
|  | 438 | N | Infantis | R |  | R |  | R |  |  |  | R |  | R |  | + | + | - |
|  | 440 | N | Infantis | R |  | R |  |  |  |  |  | R |  |  |  | + | + | - |
| February 2020 | 441 | O | Typhimurium | R |  |  |  |  | R |  |  |  |  |  |  | + | - | - |
|  | 443 | O | Typhimurium | R |  |  |  |  | R |  |  |  |  |  |  | + | + | - |
|  | 456 | O | Typhimurium | R |  | R |  |  | R |  |  |  |  |  |  | + | + | - |
|  | 458 | O | Typhimurium | R | R | R |  |  | R |  |  |  |  |  |  | + | - | - |
|  | 460 | O | Typhimurium | R |  | R |  |  | R |  |  |  |  |  |  | + | + | - |
|  | 463 | N | Infantis | R |  | R | R | R | R | R | R | R | R | R |  | + | - | - |
|  | 464 | N | Enteritidis |  |  |  |  |  |  |  |  |  |  |  |  | + | + | + |
|  | 465 | N | Kentucky | R | R |  |  |  |  |  |  |  |  |  |  | + | + | - |
|  | 471 | N | Kentucky | R | R |  |  |  |  |  |  |  |  |  |  | + | + | - |
|  | 472 | N | Kentucky | R | R |  |  |  |  |  |  |  |  |  |  | + | + | - |
|  | 474 | N | Enteritidis |  |  |  |  |  |  |  |  |  |  |  |  | - | - | + |
|  | 477 | N | Typhimurium | R |  |  | R |  |  |  |  |  | R | R | I | + | + | - |
|  | 478 | N | Kentucky | R | R |  |  |  |  |  |  |  |  |  |  | + | + | - |

N=Non-organic chicken; O= organic chicken; R= Resistance to the Antibiotic; I= Intermediate Resistance to the Antibiotic; + = The particular gene was detected; - = The particular gene was not detected.

AMP= Ampicillin; AS2= Ampicillin-sulbactam; AZT= Aztreonam; TAZ= Ceftazidime; AXO= Ceftriaxone; GEN= Gentamicin; MIN= Minocycline; NIT= Nitrofurantoin; TET= Tetracycline; TOB= Tobramycin; SXT= Trimethoprim/sulfamethoxazole.
